# The Dual Modification of PNIPAM and β-Cyclodextrin Grafted on Hyaluronic Acid as Self-Assembled Nanogel for Curcumin Delivery

**DOI:** 10.3390/polym15010116

**Published:** 2022-12-27

**Authors:** Tisana Kaewruethai, Yuan Lin, Qian Wang, Jittima Amie Luckanagul

**Affiliations:** 1 Department of Biochemistry and Microbiology, Faculty of Pharmaceutical Sciences, Chulalongkorn University, Phayathai Road, Bangkok 10330, Thailand; 2 Department of Pharmaceutics and Industrial Pharmacy, Faculty of Pharmaceutical Sciences, Chulalongkorn University, Phayathai Road, Bangkok 10330, Thailand; 3 State Key Laboratory of Polymer Physics and Chemistry, Changchun Institute of Applied Chemistry, Changchun 130022, China; 4 Department of Chemistry and Biochemistry, University of South Carolina, Columbia, SC 29208, USA; 5 Center of Excellence in Plant-Produced Pharmaceuticals, Chulalongkorn University, Phayathai Road, Bangkok 10330, Thailand

**Keywords:** hyaluronic acid, β-cyclodextrin, PNIPAM, thermoresponsive, nanogel, self-assembly, curcumin

## Abstract

Curcumin is an extract of turmeric (*Curcuma longa*) which possesses anti-inflammatory, anti-cancer and wound-healing effects and has been used as an active compound in biomedical research for many years. However, its poor solubility presents challenges for its use in drug delivery systems. A modified nanogel delivery system, with PNIPAM and β-cyclodextrin grafted onto hyaluronic acid (PNCDHA), was utilized to enhance the solubility. The polymer was characterized by NMR, and the inclusion complex between curcumin and β-cyclodextrin was confirmed by FTIR. The potential of this PNCDHA polymer complex as a drug delivery vehicle was supported by a curcumin encapsulation efficiency of 93.14 ± 5.6% and the release of encapsulated curcumin at 37 °C. At a concentration of 0.5% *w*/*v* in water, PNCDHA nanogels were biocompatible with fibroblast cell line (L929) up to a curcumin concentration of 50 µM. There was a direct concentration between curcumin loading and cellular internalization. A more detailed study of the cellular internalization of PNCDHA nanogel should be considered in order to clarify cellular delivery mechanisms and to assess how its viability as a carrier may be optimized.

## 1. Introduction

Nanogels are nanoscale drug delivery systems that are assembled from either hydrophobic or hydrophilic polymer chains [1]. The size of a particle can influence its characteristics, such as stability, cellular internalization, or blood circulation time, which may then affect its efficiency as a drug delivery platform. Nanogels have been introduced as a system that is able to provide a level of versatility of application to nanosized particles [2,3,4]. With amphiphilic characteristics and colloidal stability from the polymer network, nanogels have been proposed as solubility and stability enhancers of various types of compounds [5,6,7]. Modifications to nanogels have been introduced. Some specific modifications include the grafting of polymer backbones or additions of other groups that modify functionality. Further modifications may deliver responsiveness to stimuli such as temperature, pH, redox potential, and enzyme activity, all of which will be dependent on the local environment and/or the presence of active drugs [8,9].

Hyaluronic acid or HA, a biocompatible macromolecule found throughout the human body, has been utilized as a polymer backbone because of its safety and because its structure possesses hydroxy and carboxyl groups that facilitate the functionalization of specific moieties [10,11]. Numerous functional moieties have been utilized on the HA backbone to deliver features to the nanogels, such as stimuli responsiveness, and to increase the drug-loading efficacy. One example is pH-responsive methacrylated HA, designed to facilitate the targeted release of doxorubicin in the acidic environment around a tumor [12]. Another example is the grafting of a thiolated hydrophobic molecule onto the HA backbone, designed to deliver redox responsiveness and enhance the stability and loading capacity of curcumin and simvastatin [13]. HA has been used widely in biomedical applications as it exhibits outstanding properties related to wound healing and tissue engineering and can specifically target drugs to the CD44 receptor [14,15,16].

The backbone polymer usually incorporates a chemical moiety that provides distinct characteristics to the system and facilitates the ability to overcome the limitations of the active compound loaded in the system. One of the most common challenges relates to the solubility of active drugs, as this can limit their absorption and efficiency [17]. β-cyclodextrin (β-CD) is a nontoxic molecule that is widely used as a solubility enhancer in the pharmaceutical, food and cosmetic industries. It has a truncated cone shape that provides an inner hydrophobic space for hydrophobic drugs or active ingredients, together with a hydrophilic outer structure. Following the incorporation of hydrophobic active compounds into the interior cavity, an inclusion complex may be formed, which can enhance the overall aqueous stability of the compound [18,19].

**Figure 1:**
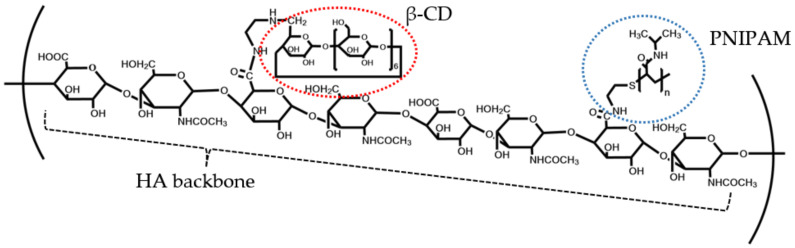
Chemical structure of PNIPAM and β-CD grafted onto HA backbone (PNCDHA).

Stimuli-responsive moieties have been incorporated into the polymer backbone in order to generate materials that display targeted drug release under specific conditions. This has the objective of increasing the specificity of the nanogels while reducing the side effects of the drugs. PNIPAM or poly(N-isopropylacrylamide) is a thermo-responsive molecule that exhibits conformational change between 32–34 °C, which may be the trigger for the release of encapsulated drugs under physiological conditions [20,21]. The modification of hyaluronic acid, as the backbone polymer, with β-cyclodextrin and PNIPAM was introduced to increase aqueous solubility and facilitate the release of curcumin under physiological conditions. The two-step modification was achieved via a simple coupling reaction using EDC/NHS, and a chemical structure of the modified polymer was shown in Figure 1. This dual-functionalized polymer was expected to form nanogels that elevate the aqueous solubility of curcumin, one example of a poorly soluble compound. This novel drug delivery system was also designed to provide biocompatibility and drug release under physiological conditions.

## 2. Materials and Methods

### 2.1. Cell Line and Culture Conditions

Murine fibroblasts (L929) were cultured in Dulbecco’s modified Eagle’s medium (DMEM) containing FBS (10%) and penicillin−streptomycin (1%) and incubated under 5% CO_2_ at 37 °C before being used in cell viability and cellular internalization studies.

### 2.2. Chemicals

β-Cyclodextrin was purchased from Glentham Life Sciences (Corsham, UK). Sodium hyaluronic acid (Mw.53kDa) was purchased from Liuzhou DALI Chemical Co., Ltd. (Guangxi, China). poly(N-isopropylacrylamide), amine-terminated (M_n_ 5500) and p-toluenesulfonyl chloride (PTSC) were purchased from Sigma-Aldrich (St. Louis, MO, USA). Ethylenediamine was purchased from Carlo Erba (Barcelona, Spain). Ammonium chloride (NH_4_Cl) was purchased from QRëC (Asia) (Kuala Lumpur, Malaysia). 1-Ethyl-3-(3-dimethyl aminopropyl) carbodiimide (EDC) and N-Hydroxysuccinimide (NHS) were purchased from Shanghai Aladdin Bio-Chem Technology Co., Ltd. (Shanghai, China). HyClone™ characterized fetal bovine serum (FBS) was purchased from Cyvita (Marlborough, MA, USA).

### 2.3. Methods

#### 2.3.1. Modification of β-Cyclodextrin-Functionalized Hyaluronic Acid (CDHA)

##### Mono-6-Deoxy-6-(P-Tolylsulfonyl)-CD, β-Tosyl-CD

Mono-6-deoxy-6-(p-tolylsulfonyl)-CD was produced following the method of a previous study [22] with some modifications. Briefly, p-toluenesulfonyl chloride (2.45 g, 22.34 mmol) and β-cyclodextrin (5.75 g, 5 mmol) were stirred in 125 mL of purified water for 2 h at room temperature. After 2 h, 25 mL of 2.5 M NaOH was added to the mixture and stirred for a further 10 min. Then, unreacted PTSC was removed using filter paper. The filtrate was adjusted to pH 8 by adding NH_4_Cl, and the solution was held at 4 °C overnight to obtain a precipitate. To remove the salt, the product was filtered and washed with cold water, followed by acetone. Holding the mixture under a high vacuum overnight then generated a fine, white powder.

##### Mono-6-Deoxy-6-Aminoethylamino-β-Cyclodextrin, β-Cyd-Ene

Mono-6-deoxy-6-aminoethylamino-β-cyclodextrin was produced using the method of a previous study [23]. β-tosyl-CD (1.50 g, 1.17 mmol) was added to 5 mL of ethylenediamine under an N_2_ atmosphere. The mixture was stirred at 60° for 12 h and cooled to room temperature. After cooling, the mixture was poured into an excess volume of EtOH, and a precipitate formed, which was filtered, then washed with EtOH and dried under reduced pressure.

##### β-Cyclodextrin-Functionalized Hyaluronic Acid (β-CDHA)

β-CDHA was prepared by a coupling reaction using EDC and NHS [24]. Briefly, sodium hyaluronate (HA, 53 kDa) (100 mg, 1.89 µmol) was dissolved in 30 mL of PBS (0.1 M, pH 7.2). After the HA had completely dissolved, EDC (88.2 mg, 0.46 mmol) and NHS (52.95 mg, 0.46 mmol) were added and stirred for 30 min at room temperature. Then, a solution of β-cyd-ene (52.32 mg, 44.54 mmol) in 10 mL of PBS was added, and the mixture was stirred for a further 24 h at room temperature. Purified β-CDHA was then generated by dialysis against a large volume of ultra-purified water for 5 days, followed by lyophilization for 72 h.

#### 2.3.2. Modification of PNIPAM Grafted β-Cyclodextrin-Functionalized Hyaluronic Acid (PNCDHA)

Poly(N-isopropylacrylamide), amine-terminated, PNIPAM-NH_2_ was functionalized on β-CDHA (5% graft) via a coupling reaction. β-CDHA (85.06 mg, 1.329 µmol) and PNIPAM-NH_2_ (46.75 mg, 8.5 mmol) were dissolved separately in 25 mL of purified water. The PNIPAM-NH_2_ solution was poured into the β-CDHA solution, followed by the addition of EDC (57.03 mg, 0.297 mmol) and NHS (34.24 mg, 0.297 mmol). The mixture was adjusted to pH 5.5 ± 0.3 using 2 M HCl and stirred for 60 min at room temperature. After 60 min, the pH of the mixture was adjusted to 7.5 ± 0.3 using 5 M NaOH and stirred at room temperature for 48 h. The polymer was then purified by dialysis against ultra-purified water for 48 h using a 12–18 kDa membrane, followed by lyophilization for 72 h. This process also led to polymer thermoresponsiveness.

#### 2.3.3. Characterization of PNCDHA Polymer and Inclusion Complex of PNCDHA and Curcumin

##### Proton Nuclear Magnetic Resonance (^1^H NMR) Spectroscopy

^1^H NMR was utilized to confirm the structure of PNCDHA. The characterization was performed at room temperature using 400 MHz (Bruker, Billerica, MA, USA). PNCDHA was completely dissolved in D_2_O prior to characterization.

##### Preparation of Self-Assembled PNCDHA Nanogel and Curcumin-Loaded Nanogel

PNCDHA nanogel was prepared by sonication in purified water for 30 min, then held at 4 °C for 48 h to allow the polymer network to settle. Curcumin was loaded into the nanogel by adding 150 μL of curcumin stock solution (10 mM in ethanol) dropwise to 1.5 mL of nanogel solution while maintaining stirring. Then the solution was incubated for 48 h at 4 °C and protected from light. The excess curcumin was removed by centrifugation at 3000× *g* for 5 min, and the supernatant was collected and held at 4 °C for further experiments.

##### Fourier Transform Infrared (FTIR) Spectroscopy

After nanogel formation and drug loading, PNCDHA nanogel and cur-loaded PNCDHA nanogel were lyophilized (Labconco, Kansas City, MO, USA). The structures of PNCDHA and the PNCDHA/curcumin inclusion complex were confirmed after a freeze-dry process using FTIR spectroscopy (PerkinElmer, Waltham, MA, USA). Spectra were recorded in the range of 400−4000 cm^−^^1^ with a resolution of 4 cm^−1^ and 16 accumulated scans (Contour GT, Bruker, Billerica, MA, USA).

##### Thermal Gravimetric Analysis (TGA)

Thermal gravimetric analysis was performed on PNCDHA using NETZSCH TG 209F3 Tarsus (NETZSCH, Selb, Germany) in a temperature range of 30–800 °C with a temperature increase rate of 10 K/min.

#### 2.3.4. Characterizations of PNCDHA Nanogel and Curcumin-Loaded Nanogel

##### Particle Size and Thermoresponsiveness of Bare and Cur-Loaded Nanogel

The particle size range of the bare PNCDHA nanogel and the cur-loaded PNCDHA nanogel was investigated using the dynamic light scattering (DLS) technique (Zetasizer nano ZS, Malvern, UK). The size and morphology of the samples were determined using a JEOL JEM-1400 transmission electron microscope (TEM) (JEOL, Tokyo, Japan) at a magnification under an accelerated voltage of 80 kV. The bare nanogel and cur-loaded nanogel samples were prepared for TEM as outlined above and carefully dropped onto a copper grid. Excess nanogel formulation was removed using filter paper before investigation.

##### Encapsulation Efficiency (EE) of PNCDHA Nanogel

Following the drug loading period of 48 h, the cur-loaded nanogel was centrifuged at 3000× *g* for 5 min to remove any unloaded curcumin. The supernatant was retained to determine the concentration of curcumin in the formulation via fluorescent intensity (ex/em wavelength 415/520 nm) using a microplate reader (CLARIOstar, BMG Labtech, Ortenberg, Germany).

Nanosep^®^ centrifugal filters, MWCO 30K (Pall, UK), were utilized to separate non-encapsulated curcumin from the nanogel. After 15 min centrifugation at 14,000× *g*, the supernatant was retained and the fluorescent intensity was measured using the same protocol as above. Encapsulation efficacy of the nanogel was calculated using the equation below.

$$\mathrm{EE}\;(\%)=\frac{\mathrm{Total}\;\mathrm{drug}\;\mathrm{amount}-\mathrm{Free}\;\mathrm{drug}\;\mathrm{amount}\;}{\mathrm{Total}\;\mathrm{drug}\;\mathrm{amount}}\text{ × }100\%$$

#### 2.3.5. In Vitro Drug Release Study

Drug release studies were performed using a dialysis method that incorporated a 12–14 kDa dialysis bag. PBS (0.01 M) containing 30% ethanol (*v*/*v*) (final pH 7.4) was used as the sink medium, shaken at 100 rpm, at 37 °C, for 24 h [25]. At predetermined time points, 500 µL of medium was withdrawn and replaced with the same amount of fresh-release medium. Curcumin concentration in the samples was calculated using a fluorescence calibration curve. The resulting fluorescent intensity (ex/em wavelength 415/520 nm) was recorded, and the cumulative in vitro release of curcumin was determined as a function of the incubation time.

#### 2.3.6. Cytotoxicity Experiment

Cytotoxicity of the bare PNCDHA nanogels (with polymer concentrations ranging from 0.125–1% in PBS) and the cur-loaded nanogels (with curcumin concentrations in the range of 10–100 µM) was performed in L929 cells using the 3-(4,5-dimethylthiazol-2-yl)-2,5 diphenyl tetrazolium bromide (MTT) assay. L929 cells were seeded into 96-well plates (1.5 × 10^5^ cell/well) and incubated under 5% CO_2_ at 37 °C for 24 h. The cultured medium was removed, and the cells were washed with sterile PBS. 100 µL of Dulbecco’s modified Eagle’s medium (DMEM) containing penicillin−streptomycin (1%) was added, followed by 50 µL of various concentrations of PNCDHA nanogel and cur-loaded PNCDHA nanogel. After 24 h incubation, the cells were washed with PBS, followed by the addition of 10% MTT solution in PBS (100 µL) and incubated for an hour. Then the MTT solution was replaced by 100 µL of DMSO to dissolve formazan crystals, and the absorbance was measured at 570 nm. The % cell viability was calculated using the equation below.

$$\%\mathrm{Cell}\;\mathrm{viability}=\frac{\mathrm{absorbance}\;\mathrm{of}\;\mathrm{treated}\;\mathrm{cell}\;}{\mathrm{absorbance}\;\mathrm{of}\;\mathrm{treated}\;\mathrm{cell}}\text{ ×}100$$

#### 2.3.7. Cellular Internalization Study

L929 cells were seeded into 96-well plates at a density of 1.5 × 10^5^ cells/well. After 24 h incubation under 5% CO_2_ at 37 °C, the culture medium was removed, and the cells were washed with PBS. 100 µL of fresh DMEM was added, then 50 µL of cur-loaded PNCDHA nanogel was added to generate final curcumin concentrations of 10, 20, 30, 40 and 100 µM. The treated cells were incubated for 2, 4, 6 and 24 h, and the cellular internalization of the cur-loaded PNCDHA nanogels was assessed. Cells treated with the same range of concentrations of curcumin stock solution in DMSO were used in parallel as a positive control. At each incubation time point, cellular internalization of cur-loaded nanogel and curcumin in DMSO was observed under an Olympus IX51 inverted fluorescent microscope (Olympus, Tokyo, Japan), with excitation at 520 nm, to assess curcumin fluorescence and bright field mode to assess cell morphology.

##### Flow Cytometry

L929 cells were seeded into 48-well plates at 1.5 × 10^5^ cells/well and incubated for 24 h. After reaching 80–90% cell confluence, the culture medium was removed, and the cells were washed with PBS before the addition of 100 µL of DMEM, plus 50 µL of 10, 20, 30, 40 and 50 µM of cur-loaded PNCDHA nanogel. Cells treated with bare nanogel and curcumin stock solution in DMSO with the same concentrations of curcumin were performed in parallel as negative and positive controls, respectively. The treated cells were incubated under 5% CO_2_ at 37 °C for 2 h. The cells were washed with PBS twice, then TrypLE™ Express (150 µL) (Thermo Fisher Scientific, Waltham, MA, USA) was added to each well to harvest the cells. Cells were incubated for 5 min and centrifuged for 5 min at 500× *g*. 1× PBS with FBS (1%) was added to replace TrypLE™ Express and to wash the cells. The cells were centrifuged again and resuspended in 200 µL of PBS with FBS (1%) before measuring the fluorescent signal by flow cytometry.

## 3. Results and Discussion

### 3.1. Structural Characterizations of PNCDHA Polymer

#### 3.1.1. NMR

**Figure 2:**
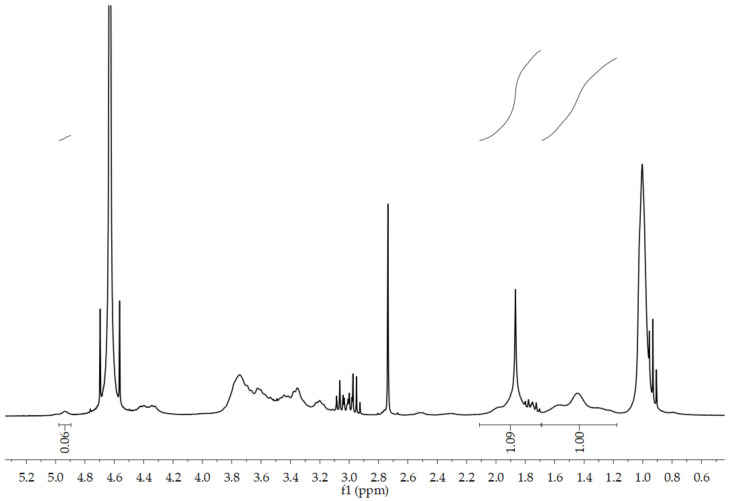
^1^H NMR spectrum of PNCDHA.

The structure of PNCDHA was confirmed by ^1^H NMR spectrum, as shown in Figure 2. The spectrum shows the main peaks from three components of the PNCDHA polymer. The peak at ~2 ppm confirmed the N-acetyl group of HA, while the peaks at 3.8 ppm and 1.0 ppm indicated the grafting of PNIPAM onto the HA backbone [26,27]. The peak around 2.7 ppm shows the protons of two aliphatic carbons on β-CyD-ene, which confirms the functionalization of β-CD on the HA structure [28]. In addition, the H1 proton of β-CD was confirmed by the presence of the small peak at ~5 ppm, indicating the truncated cone structure. The multiplet at 3.0–4.0 ppm confirmed the presence of β-CD and HA structures [24,29]. The degree of substitution of β-CD on the HA backbone was calculated using the comparison between the integrated peak area of the H1 proton of β-CD and the N-acetyl peak of HA. For PNIPAM, the degree of substitution was determined using the calculation of Luckanagul JA [30].

#### 3.1.2. FTIR

**Figure 3:**
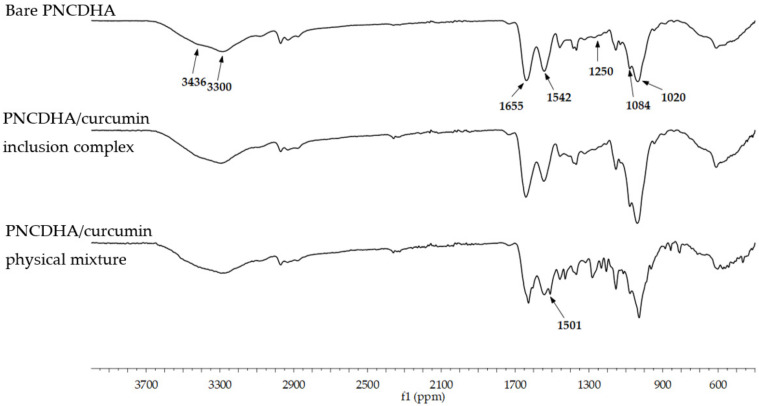
FTIR spectra of bare PNCDHA, inclusion complex of PNCDHA/curcumin and PNCDHA polymer physically mixed with curcumin.

The FTIR spectra of PNCDHA (as shown in Figure 3) indicated the characteristic peak of carbohydrates at 944–1155 cm^−^^1^, which included the characteristic peak of HA at 1084 cm^−^^1^ and the C-N peak of amine at 1250 and 1020 cm^−^^1^ [31]. The characteristic curve at 3385 cm^−^^1^, which represented symmetrical and asymmetrical stretching of the β-CD hydroxyl groups, confirmed the structure of β-CD in the HA backbone, likewise the peak at 2925 cm^−^^1^ [32]. The primary amine peak of PNIPAM was shown at 3436 and 3300, together with amide I,II peaks at 1655 and 1542, and the peak of the methyl group at 1378, all of which confirmed the success of PNIPAM functionalization on the HA backbone [28]. The inclusion complex between β-cyclodextrin on PNCDHA and curcumin was observed in the cur-loaded spectrum as the characteristic band of curcumin at 1501 cm^−^^1^ was not evident in the spectrum of cur-loaded PNCDHA [33]. The spectrum of the physical mixture between PNCDHA and curcumin displayed the characteristic curcumin peak. Finally, the spectra of the PNCDHA nanogel and inclusion complex showed no significant differences, which confirmed the inclusion complex structure between β-cyclodextrin on PNCDHA and curcumin.

#### 3.1.3. TGA

**Figure 4:**
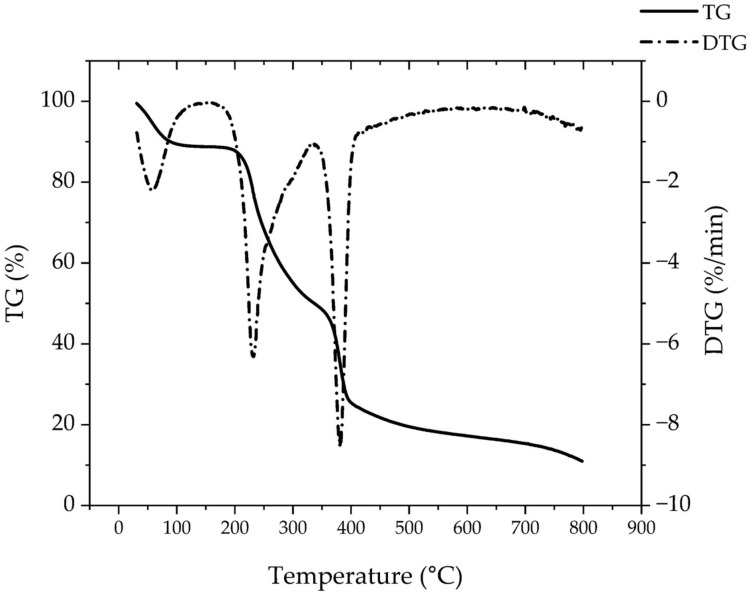
TGA graph of PNCDHA polymer.

The TGA graph of the PNCDHA polymer (Figure 4) demonstrated three phases of degradation that indicated three components in the PNCDHA polymer. The first, around 90–100 °C, resulted from the evaporation of water from the surface of the polymer [34,35]. The second, at 200–230 °C, accompanied by a weight loss of 38.5%, may possibly be a consequence of the partial degradation of HA [36,37,38]. The third phase, at 360–380 °C, is related to β-CD [35,36,39]. The third phase may also include the degradation of PNIPAM-NH_2_, which has been reported around 300 and 320–430 °C [40,41].

### 3.2. Characterization of PNCDHA Nanogels

**Figure 5:**
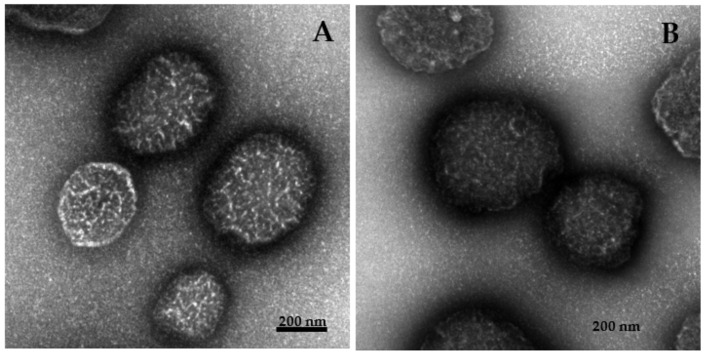
TEM images of bare PNCDHA nanogel (**A**) and cur-loaded PNCDHA nanogel (**B**).

After the preparation of PNCDHA nanogels and curcumin loading, the sizes of bare and cur-loaded PNCDHA nanogels were observed using dynamic light scattering (DLS) and TEM. The data from DLS revealed that bare and cur-loaded nanogels have a diameter of 562 ± 46 and 772 ± 45 nm, respectively. It is possible that the increase in the diameter of cur-loaded PNCDHA nanogels was a consequence of the curcumin content. Under TEM, the diameter of the bare and cur-loaded nanogels was around 200 nm (Figure 5A,B). This is smaller than the hydrodynamic diameter of the nanogels, which may be a consequence of water diffusion into the PNCDHA nanogel. PNIPAM is a thermoresponsive material, so PNCDHA nanogels were expected to display physicochemical changes during temperature fluctuations. The results from DLS confirmed the thermoresponsive behavior of bare and cur-loaded PNCDHA nanogels, which had a larger hydrodynamic diameter at higher temperatures. In addition, the lower critical solution temperature (LCST) of the nanogels was found to be 32–34 °C, which could prove beneficial in the application of these nanogels as controlled drug release carriers at specific temperatures.

### 3.3. Encapsulation Efficiency and Drug Release Studies

#### 3.3.1. Encapsulation Efficiency

The encapsulation efficiency of cur-loaded PNCDHA was determined to be 93.14 ± 5.6%. The high level of encapsulation efficiency was a consequence of the hydrophilic group of PNIPAM. This efficiency was boosted by the accommodation of the curcumin within the β-CD cavity, possibly as a consequence of van de Waals force, hydrophobic interactions and hydrogen bonding [42].

#### 3.3.2. Drug Release Studies

**Figure 6:**
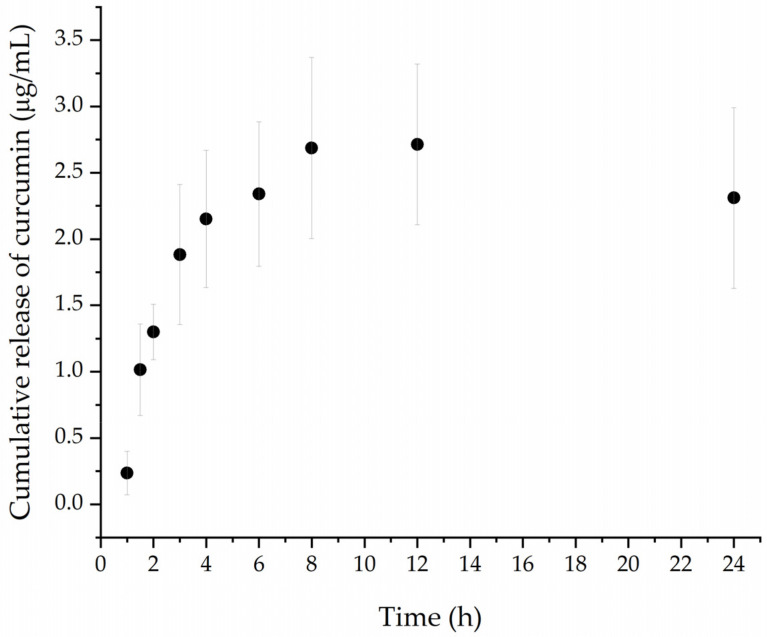
Concentration of curcumin release from PNCDHA nanogel (ug/mL) in PBS-ethanol media under 37 °C.

The release rate from nanogels is an important factor in their efficacy as drug carriers and is also associated with the stability of the drug in the formulation under specific conditions [43]. In this study, drug release was performed under physiological conditions for 24 h. In Figure 6, the results revealed that PNCDHA nanogels exhibited rapid curcumin release during the first two hours, after which a slower rate of release was observed. The curcumin release reached its maximum at 12 h. The initial rapid release is a likely consequence of the large difference in curcumin concentration within the nanogels and in the release medium [44]. Moreover, the rate of curcumin release could be facilitated by the thermoresponsiveness of the nanogels. As the size of the nanogels was found to increase significantly above the LCST, this could induce a conformation change which, together with a change of hydration state and volume, may result in a higher rate of curcumin release under the study conditions at 37 °C [45]. The relatively low release rate of the hydrophobic curcumin could also be a consequence of its preference for the hydrophobic cavity of the β-CD. The apparent decrease in curcumin concentration beyond 12 h could be a consequence of its degradation in the buffer solution under the study conditions [46,47].

### 3.4. Cytotoxicity Test

**Figure 7:**
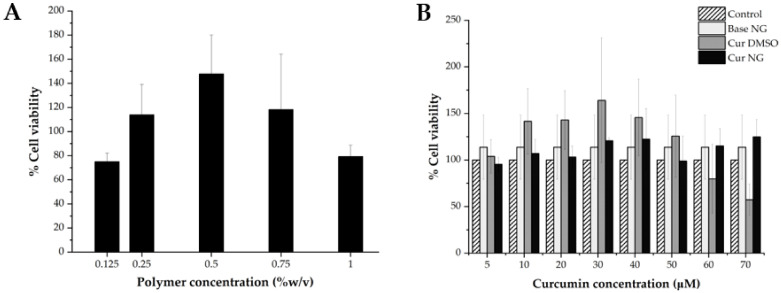
% cell viability of L929 cells after being treated by bare PNCDHA nanogel in different polymer concentrations (**A**) and cur-loaded PNCDHA nanogel with various curcumin concentrations (**B**) for 24 h.

The data from cytotoxicity testing of PNCDHA nanogels showed that 0.25%, 0.5% and 0.75% nanogels exhibited no toxicity on L929 cells after 24 h treatment (Figure 7A). The highest % cell viability was found in 0.5% nanogels, which could be a consequence of the cell proliferation enhancement properties of HA. Meanwhile, the reduced % cell viability in the 1% PNCDHA sample could possibly be confirmation that high concentrations of HA can decrease cell viability after 24 h incubation, as has been reported elsewhere [48,49]. 

For cur-loaded PNCDHA nanogels, the data in Figure 7B showed a higher % cell viability of L929 in the formulations that contained 10–50 µM curcumin, compared to the control, and the highest percentage of cell viability was found at 40 µM curcumin loading. Additionally, the results revealed that, across the range of 5–50 µM curcumin, the % cell viability of L929 was lower than for curcumin in DMSO. This is possible because penetration enhancement from DMSO directly facilitated the cellular internalization of curcumin, leading to an increase of % cell viability at 5–50 μM of curcumin [50,51]. This result is supported by the bright field images of the L929 cell after 24 h incubation with cur-loaded nanogels. These images revealed the formation of apoptotic bodies in cur-loaded nanogels that could indicate signs of cell death. The higher anti-proliferative effect of curcumin/β-cyclodextrin inclusion complex compared to curcumin alone was reported in the work of Ja’far M.H. et al. [52]. The decrease in cell viability of the cur-loaded PNCDHA nanogel observed in this study requires further investigation.

### 3.5. Cellular Internalization Study

**Figure 8:**
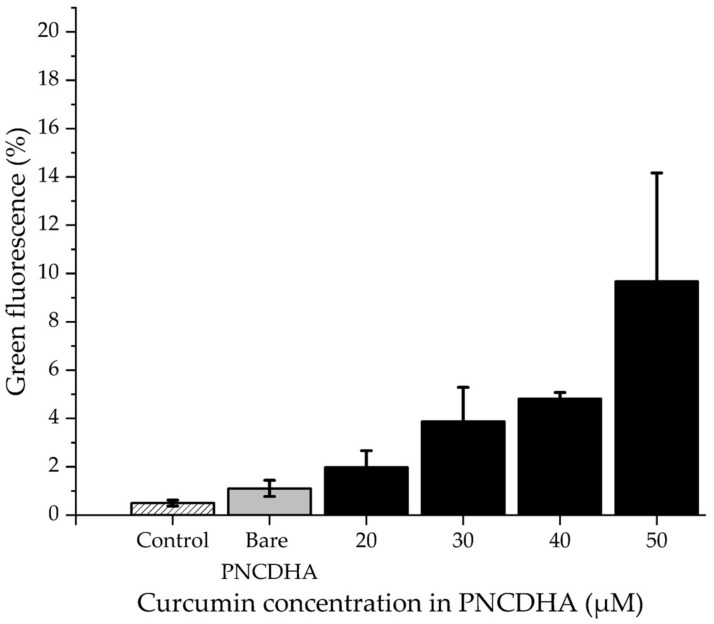
The data from flow cytometry shows percentage of green fluorescence detected in PNCDHA containing various concentrations of curcumin. (Each column represents means. Error bars are standard error of means (SEM)).

After 2 h of incubation, the cellular internalization of the cur-loaded PNCDHA nanogel was assessed by flow cytometry. The results demonstrated an increase in the detection of intracellular green fluorescence from curcumin in the PNCDHA nanogel with increasing concentrations of encapsulated curcumin (Figure 8). These results demonstrate a relatively greater amount of intracellular green fluorescence when compared to cells treated with curcumin in DMSO (data not shown). The cur-loaded PNCDHA nanogels in this study were found to be 700 nm in diameter. Some previous studies have reported that the size of nanoparticles can influence cellular internalization, with the optimum size believed to be 50–200 nm [53]. A further study determined that the cellular internalization of particles that can be influenced by temperature has an upper size limit of 500 nm at 37 °C. Moreover, the 500 nm particles were principally located at the cell periphery, while the smaller particles of 50, 100 and 200 nm were distributed throughout the cell [54]. 

## 4. Conclusions

In this study, it has been established that dual modification of PNCDHA polymer can be performed via a simple coupling reaction. After polymer modification, its chemical structure was confirmed via NMR and FTIR. The inclusion complex of curcumin and β-cyclodextrin was investigated using FTIR spectra. Then, the size of the nanogels was observed using TEM and DLS, indicating a sub-micron size of bare and curcumin-loaded nanogel. The thermoresponsiveness of nanogels was investigated, and the results demonstrated the LCST of the nanogel was 34–36 °C, which may impart some benefits for drug delivery under physiological conditions. According to its thermosensitive property, curcumin was released from cur-loaded nanogel at 37 °C for 12 h. Finally, curcumin-loaded nanogel exhibited a degree of biocompatibility and demonstrated internalization into the fibroblast cell, L929. All the data indicated that the properties of PNCDHA nanogel may show some promise as a drug delivery system.

## Data Availability

Not applicable.
